# Supplementary material for: Genome Profiling of H3k4me3 Histone Modification in Human Adipose Tissue during Obesity and Insulin Resistance
Source: Biomedicines. 2021 Sep 30;9(10):1363. doi: 10.3390/biomedicines9101363 (PMC8533428; doi:10.3390/biomedicines9101363)
Supplement: Supplementary file 1 [file Supplementary figures and Tables .pdf › Supplementary figures and Tables .pdf]

SUPPLEMENTARY

Table S1. Anthropometric and biochemical variables.

	NOb	Ob.LIR	Ob.HIR
**Age**	49.75±7.93	45.75±9.35	48.00±10.73
**Gender (M/F)**	2 / 2	0/4	3 / 1
**Diabetes**	0	0	1
**Statins**	0	0	1
**Metformin**	0	0	1
**Waist**	85.75±8.84 [a]	133.75±11.61 [b]	154.00±18.20 [b]
**BMI**	24.04±2.09 [a]	51.34±0.00 [b]	52.6±0.50 [b]
**Glucose**	104.75±9.57	99.5±12.87	122.75±26.31
**Insulin**	12.73±7.88 [a]	14.27±3.28 [a]	36.657±7.57 [b]
**HOMA-IR**	3.30±2.06 [a]	3.53±1.11 [a]	10.80±1.55 [b]
**Tg**	123.25±45.96	147.75±39.81	112±25.78
**Chol**	229.25±36.48	209±42.24	174.5±39.06
**HDL-Cho**	55.25±23.30	42.75±8.26	45.50±5.80
**LDL-Cho**	156.25±47.89	133.85±44.82	105.80±33.72

Abbreviations: Body mass index (BMI); Homeostatic model assessment of insulin resistance (HOMA-IR); triglycerides (Tg); total cholesterol (Chol); high-density lipoprotein cholesterol (HDL-Cho); low-density lipoprotein cholesterol (LDL-Cho). Different letters mean differences among groups (pval<0.05).

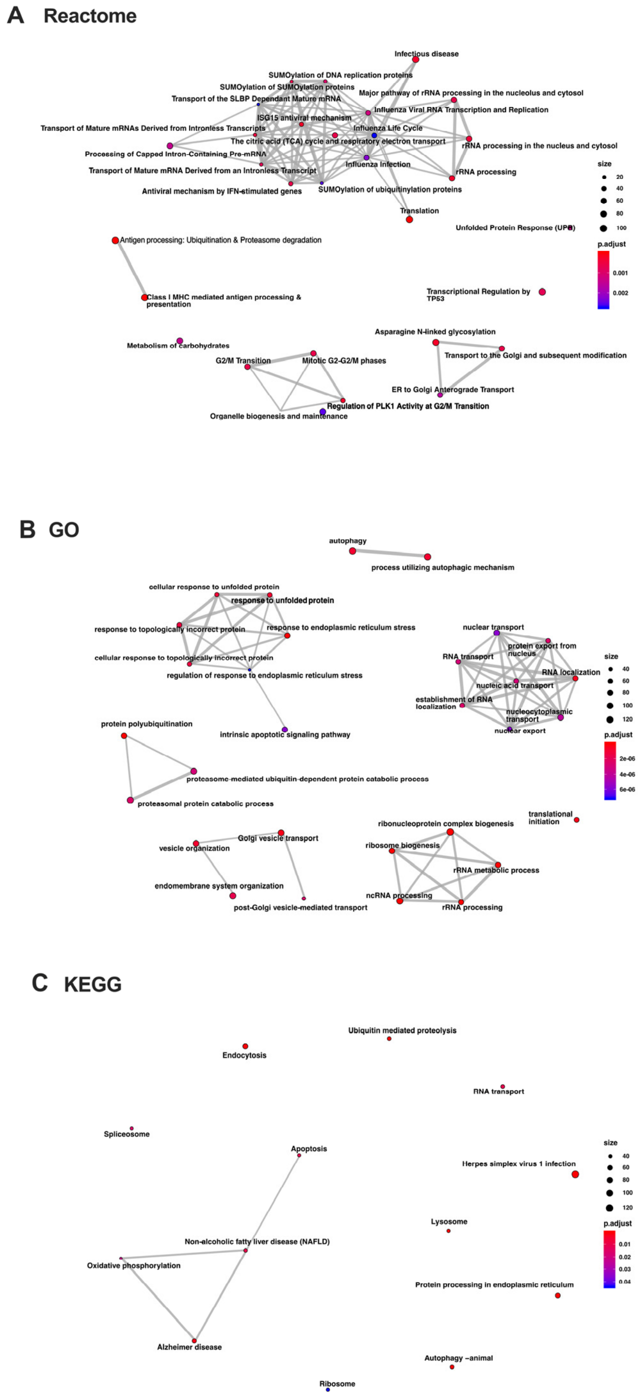

Figure S1. The top significantly H3K4me3 enriched pathways analyzed by Reactome (A), GO biological function (B) and KEGG (C) databases.

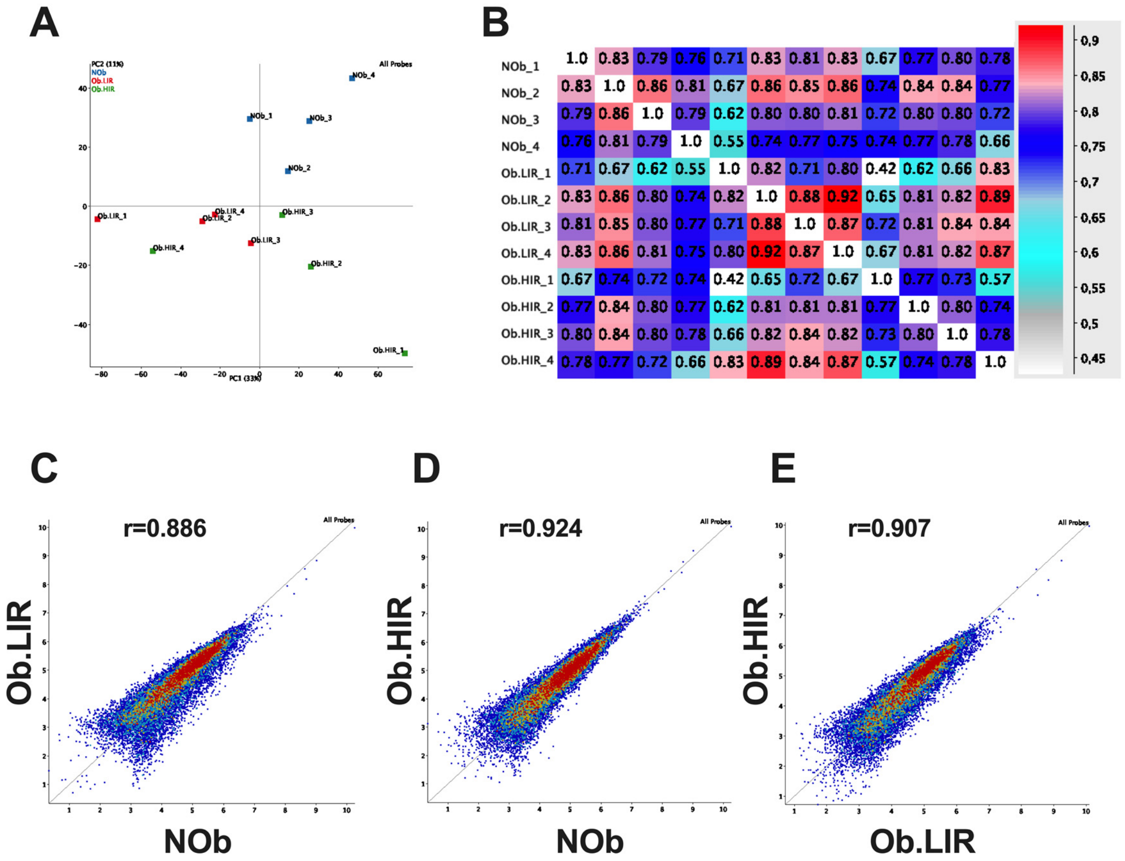

Figure S2. Store similarity for H3K4me3 enrichment among study participants. PCA plot (A) and correlation matrix (B). H3K4m3 store similarity among the 3 groups (C, D and E).

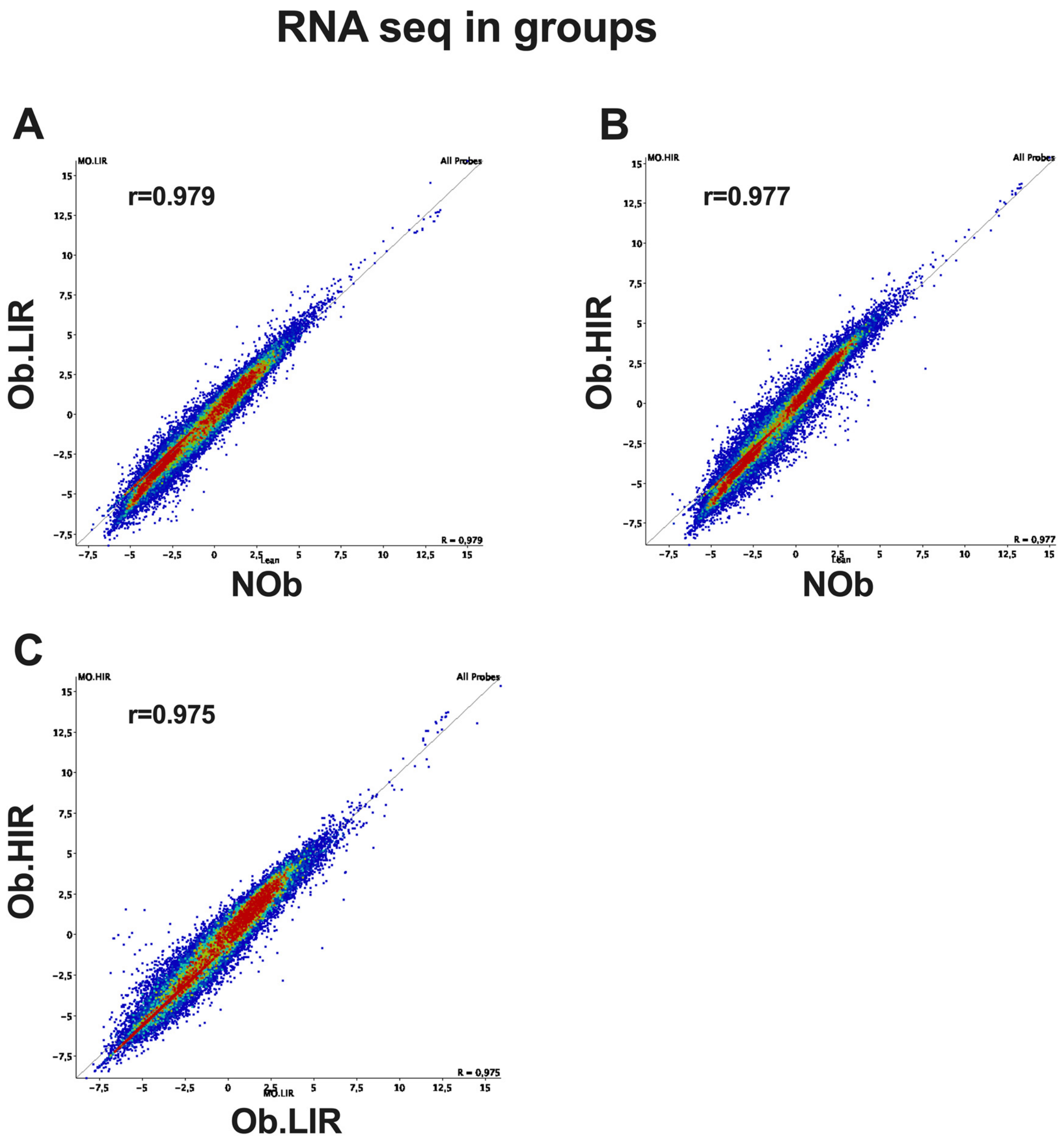

Figure S3. Store similarity for mRNA expression among study groups.
